# TRENDS IN COMMUNITY-BASED CARE STAFFING LEVELS IN OREGON: DIFFERENCES BY FACILITY CHARACTERISTICS

**DOI:** 10.1093/geroni/igad104.1099

**Published:** 2023-12-21

**Authors:** Sarah Dys, Ozcan Tunalilar, Paula Carder

**Affiliations:** Portland State University, Portland, Oregon, United States; Portland State University, Portland, Oregon, United States; Portland State University Institute on Aging, Portland, Oregon, United States

## Abstract

The COVID-19 pandemic re-centered the workforce crisis across long-term care settings in the U.S. Variation in regulatory requirements, industry practices, staff training, and lack of longitudinal data introduces complexity in understanding workforce trends specific to assisted living and residential care (AL/RC). Pooling five waves of the Oregon Community-Based Care study, we examine trends in AL/RC care staff levels and associations with facility characteristics: license type, Medicaid census, profit status, and rurality. From 2017-2022, 548 unique facilities participated in the study, 55% contributing to four to five waves. The estimated average care staff-to-resident ratio was 0.79 (median= 0.67), increasing between 2017 (0.74) and 2022 (0.87). The average staffing level (care hours per resident per day; hours:minutes) was 3:28 (median= 2:58). We estimated ordinary least squares regression models with robust standard errors, controlling for facility-level characteristics and year. On average, staffing levels were higher by 1:38 in memory care (MC) and 0:42 in nonprofit settings. Compared to non-Medicaid settings, overall staffing levels were about 1:00-1:30 lower in mixed-payer settings, and 2:00 higher in settings providing specialty care (e.g., behavioral health, bariatric) at increased Medicaid reimbursement rates. Licensed nursing staff levels were slightly higher in MC, nonprofit, and special contract Medicaid settings. Certified nursing/medication aide levels were 0:30 higher in nonprofit settings. There were no differences in caregiver levels by profit status or rurality, though mixed-payer setting levels were lower by 0:32-0:44. Reduced occupancy during the pandemic resulted in higher staffing levels, though findings suggest continued disparities in staff mix by facility characteristics.

